# Hemorrhagic Rupture of a Hepatic Adenoma in a Postmenopausal Female: A Case Report

**DOI:** 10.7759/cureus.106659

**Published:** 2026-04-08

**Authors:** Rebecca Corallo, Carrie Watson

**Affiliations:** 1 Medicine, Edward Via College of Osteopathic Medicine, Spartanburg, USA; 2 Trauma Surgery, Piedmont Medical Center, Rock Hill, USA

**Keywords:** hemoperitoneum, hemorrhagic rupture, hepatic adenoma, oral contraceptive pills, postmenopausal female

## Abstract

Hepatic adenoma (HA) is a rare benign liver lesion most commonly observed in females of reproductive age using oral contraceptive pills (OCPs). Though typically asymptomatic, HA may lead to complications such as hemorrhage or malignant transformation. Current literature highlights an increased risk of hemorrhage in larger lesions among premenopausal females and transformation to hepatocellular carcinoma in postmenopausal females. This report discusses a case of a 58-year-old postmenopausal female who presented with a sudden onset of back pain, syncope, nausea, and vomiting. CT imaging revealed moderate- to large-volume intraperitoneal fluid concerning for hemoperitoneum and a right hepatic mass, prompting urgent surgical intervention. The patient was ultimately diagnosed with a hemorrhagic rupture of a 3.8-cm HA. Postoperatively, the patient was advised to discontinue OCP use and undergo repeat imaging in three months. Subsequent scans revealed a minor increase in size and a slight elevation in alpha-fetoprotein. The patient has been referred to oncology for further recommendations. This case aims to increase recognition of HA complications, specifically hemorrhage as a potentially fatal presentation, which can present atypically, even in postmenopausal patients.

## Introduction

Hepatic adenoma (HA) is often an incidental finding but can lead to serious complications. HA is most common in females, with a 4:1 ratio compared to males. Oral contraceptive pill (OCP) use is directly associated with HA formation, with the annual incidence in this group being three to four per 100,000. This is significantly greater than the annual incidence of HA among patients not taking OCPs, which is one per 100,000 [1-3]. Risk factors of HA include exposures leading to an overall increased level of estrogen and androgen. This includes but is not limited to OCP use, exogenous steroids, obesity, and metabolic syndromes [1-4]. HA is most often identified through incidental imaging, as the disease process is commonly asymptomatic [2]. Symptoms typically arise in the setting of complications and present as abdominal pain, pallor, or weak pulses [3,5]. HA management is guided by the patient's sex and lesion size. It is recommended that HA be resected in males regardless of age. Females follow the recommendation to resect lesions greater than 5 cm and to manage smaller lesions conservatively [3]. There are two main complications of HA. The first and most likely is hemorrhage. This occurs in 27% of cases and is more commonly associated with lesions greater than 5 cm. Malignant transformation is the second complication and is more commonly observed in males, in lesions greater than 5 cm, and in postmenopausal females [3,6-8]. The purpose of this case report is to highlight potential complications of HA, including malignant transformation and, in this case, hemorrhage as a potentially fatal presentation, even in patients who appear to have a lower risk profile.

## Case presentation

**Figure 1:**
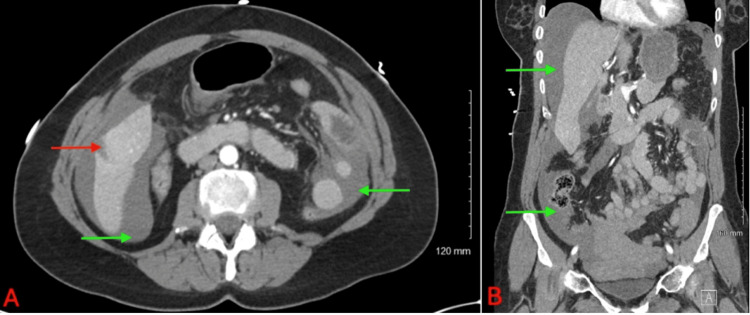
CT abdomen and pelvis (A) Axial CT imaging. Red arrow pointing at a 4 cm nodule in the right lobe of the liver. Green arrows demonstrating diffuse hyperdense peritoneal fluid, 32 HU in the mid to upper abdomen, and 67 HU in the pelvis. (B) Coronal CT imaging. Green arrows demonstrating diffuse hyperdense peritoneal fluid. CT: computed tomography

**Figure 2:**
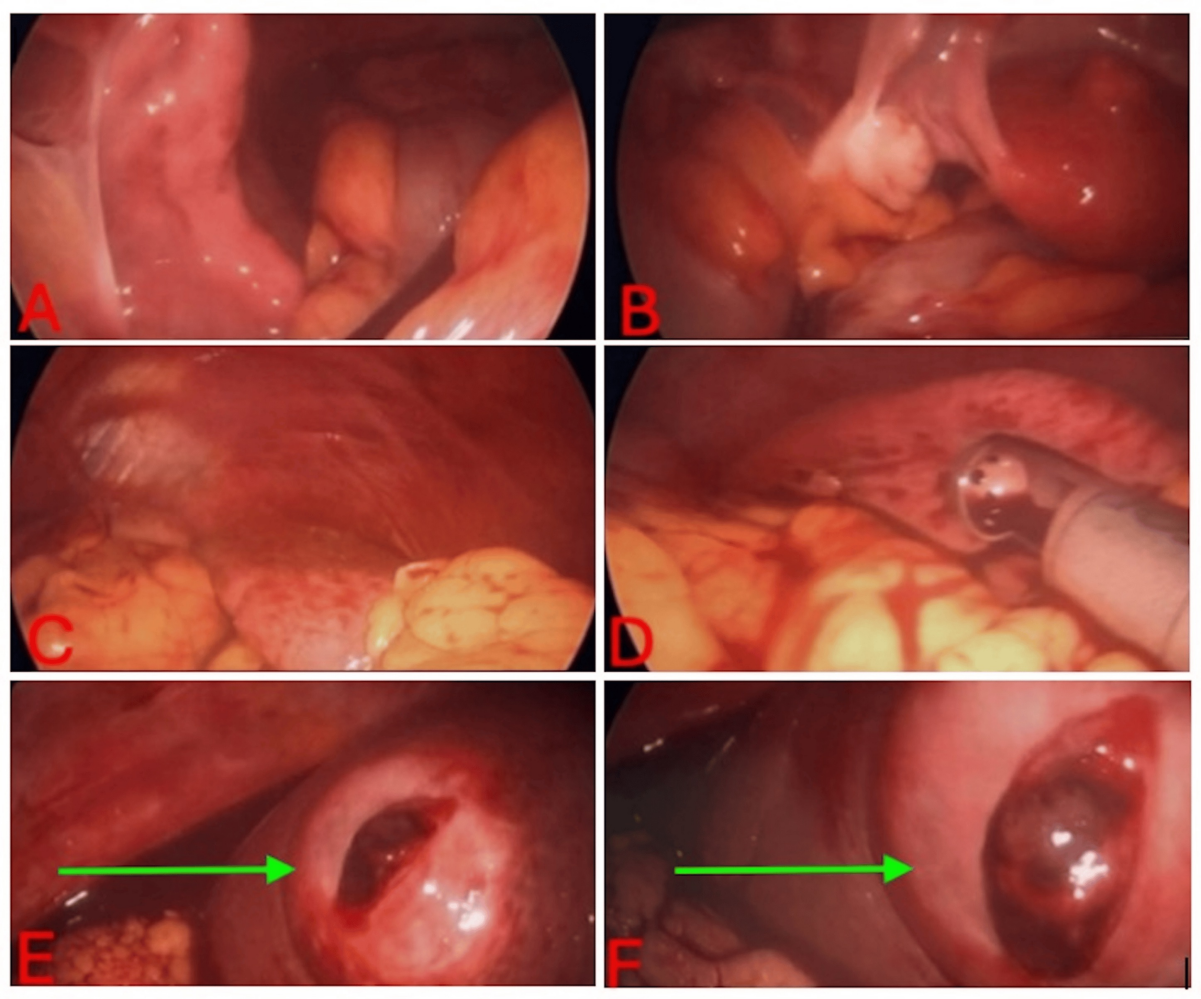
Intraoperative laparoscopic imaging (A-D) No evidence of active bleeding or injury in the upper or lower abdominal cavity. (E, F) Clotted, non-actively bleeding mass on the right hepatic lobe as demonstrated by the green arrow.

A 58-year-old postmenopausal female with a normal body mass index presented to the emergency department at Piedmont Medical Center - Rock Hill, South Carolina, in September of 2024, with a sudden onset of right-sided back pain, followed by two episodes of syncope accompanied by nausea, vomiting, and diarrhea. She denied fevers, chills, urinary symptoms, or vaginal bleeding. Her medical history was notable for OCP use for menopausal symptoms and an appendectomy in 2022, with no other significant medical or surgical history. Emergency medical services reported that the patient was initially hypotensive. They administered nonsteroidal anti-inflammatory drugs, antiemetics, and normal saline. Upon clinical examination, the patient did not display signs of acute distress; she appeared pale and was cooperative. There were no obvious deformities, her heart rhythm was regular, and she had non-labored breathing without any audible wheezing. Peripheral pulses were normal and equal. There was no back pain, and she had a normal range of motion in her extremities, with no skin rashes noted. Her abdomen was diffusely tender without signs of peritonitis; the abdomen was soft and non-distended. The patient was mildly tachycardic, with a heart rate in the 90s to 100s beats per minute. Further diagnostic workup, including CT imaging, revealed hemoperitoneum with attenuation values of 32 Hounsfield units (HU) in the mid-to-upper abdomen and 67 HU in the pelvis, along with a 4-cm hyperdense hepatic nodule in the right lobe (Figure 1). Laboratory evaluation was significant for a hemoglobin level of 8.6 g/dL (Table 1). These findings led to a differential diagnosis to include hemorrhagic ovarian cyst and metastatic disease with a new onset of ascites. A diagnostic laparoscopy was performed to evacuate the hemoperitoneum and evaluate for a gynecologic source of bleeding versus other sources of bleeding. Intraoperative findings revealed no evidence of intra-abdominal injury or ruptured ovarian cysts; however, a significant volume of hemoperitoneum with a large pelvic clot was noted. Evacuation of roughly 2200 cc of hemoperitoneum allowed for visualization of a ruptured lesion on the anterior superior aspect of the right hepatic lobe (Figure 2). To minimize the risk of rebleeding, the overlying clot was left intact, and an absorbable hemostatic powder was applied to promote local hemostasis.

**Table 1:** Laboratory results: hematology basic and chemistry comprehensive

Test	On admission	Immediately postoperative	Postoperative day 1	Reference	Units
White blood cell count	18.1	10.8	13.8	4.8-10.8	X10(3)/mcL
Hemoglobin	8.9	6.6	7.7	12.0-16.0	g/dL
Hematocrit	38	19.6	23.1	37.0-47.0	%
Platelet count	220	182	165	150-400	X10(3)/mcL
Sodium	137	-	138	133.0-145.0	mmol/L
Potassium	4.6	-	3.9	3.50-5.00	mmol/L
Chloride	104	-	106	95-105	mmol/L
CO2	21	-	26	22.0-32.0	mmol/L
Glucose	145	-	120	70-105	mg/dL
BUN	21	-	13	8-26	mg/dL
Creatinine	1.3	-	0.7	0.6-1.1	mg/dL
AGAP	17	-	10	8-16	mmol/L
Albumin	3.7	-	3.7	3.5-4.8	g/dL
Bilirubin total	0.3	-	0.3	0.3-1.2	mg/dL
Alkaline phosphatase	29	-	29	39-117	IU/L
Aspartate transaminase	17	-	17	15-41	IU/L
Alanine transaminase	13	-	13	14-54	IU/l
Lactic acid	6.58	-	1.37	0.63-1.99	mmol/L

**Figure 3:**
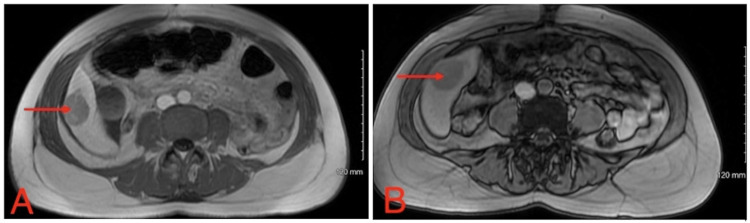
MRI abdomen (A) MRI abdomen three days postoperative. Red arrow identifying a 3.8 cm mass of the right liver consistent with HA. (B) MRI abdomen three months postoperative, with the red arrow depicting a 3.8 cm right liver lesion. MRI: magnetic resonance imaging, HA: hepatic adenoma

A basic hematology panel obtained immediately postoperatively revealed that the patient’s hemoglobin had dropped to 6.6 g/dL and subsequently increased to 7.7 g/dL on postoperative day 1 following transfusion of 1 unit of packed red blood cells (Table 1). Additionally, the patient’s elevated lactic acid level of 6.58 mmol/L and creatinine of 1.3 mg/dL on admission improved to 1.37 mmol/L and 0.7 mg/dL, respectively, on postoperative day one. Interventional radiology was consulted, and an abdominal MRI was performed to evaluate the lesion and assess the need for possible angioembolization if bleeding were to recur. Imaging revealed a 3.8-cm right hepatic mass, most consistent with a HA (Figure 3A). Given the lesion’s size, location, and absence of active bleeding, the interventional radiology team elected not to proceed with embolization.

The patient was discharged three days postoperatively, with a follow-up MRI scheduled for three months later. She was informed that her use of OCPs likely contributed to the development of the adenoma and was advised to discontinue them.

A repeat MRI performed three months postoperatively demonstrated a lesion of unchanged size despite cessation of OCPs (Figure 3B). An additional follow-up MRI six months postoperatively revealed a minor increase in size with a slight increase in alpha-fetoprotein. The patient has been referred to oncology for further management.

## Discussion

This case illustrates a major complication, hemorrhagic rupture, associated with an uncommon condition, HA, in a postmenopausal female. HA is a benign tumor linked to several risk factors. It is thought to be primarily hormone driven, as reflected by its high prevalence in patients using estrogens, such as OCPs, and androgens or anabolic steroids [1-4]. It has been found that the prevalence of HA increases 30-40-fold in patients using OCPs and is most common in women of childbearing age. Risk has also been noted to rise with cumulative androgen exposure, to the extent that individuals suspected of anabolic steroid use should be monitored for associated hepatic complications. Additional risk factors of HA include metabolic syndromes and obesity, likely related to increased interleukin-6 production by adipocytes, as well as glycogen storage diseases [5].

HAs are typically asymptomatic and are often diagnosed incidentally in young females through imaging studies or after complications, such as hemorrhage, arise [2-3]. If symptoms present, they can range from acute or chronic right upper quadrant or epigastric pain and anemia to more severe presentations, including circulatory shock following rupture of the HA [5]. Approximately 30-40% of patients report abdominal discomfort, and 2-4% have a palpable abdominal mass, with pain most commonly occurring after spontaneous rupture of the lesion, as seen in this patient [9].

Imaging modalities used in the diagnosis of HA include ultrasound, contrast CT, and MRI. Ultrasound is typically the initial study and may demonstrate an isoechoic lesion or a hypoechoic lesion in patients with hepatic steatosis; an anechoic center may be identifiable if prior hemorrhage has occurred [2,4-5]. CT and MRI are highly sensitive and specific for identifying HA [4]. On CT, lesions may appear hypodense when steatotic or hyperdense in the setting of acute hemorrhage, as seen in this case [5]. MRI is the preferred diagnostic modality due to its ability to characterize and classify HA subtype [4,10]. If imaging findings are inconclusive, a biopsy may be considered as an alternative diagnostic approach [1,6]. However, this approach remains controversial because of the increased risk of complications, including bleeding and hemorrhage [9].

HAs exhibit two primary complications: hemorrhagic rupture and malignant transformation. These complications often occur as the tumor enlarges and outgrows its blood supply [2]. Hemorrhage is a particularly serious complication and may be life-threatening, specifically when rupture leads to hemodynamic instability from intraperitoneal bleeding; however, in some cases, it may be partially contained by the liver capsule [10]. Rupture is most commonly observed in premenopausal females aged 28-37, particularly in lesions larger than 5 cm and in the presence of risk factors such as obesity, steroid use, and OCPs [2,6,11]. Although rare, malignant transformation of HA into hepatocellular carcinoma has been reported. This complication is more commonly seen in postmenopausal females aged 48-68, in tumors exceeding 5 cm, in patients with a history of steroid use, and in male patients, with less frequent association in those using OCPs [3,6-8].

As demonstrated in this patient, HA management is typically conservative, emphasizing risk factor modification and observation, particularly for lesions smaller than 5 cm [2,6,11]. This includes cessation of OCPs, weight loss, dietary modification, and bariatric surgery when appropriate [2]. Surgical intervention is recommended for lesions exceeding 5 cm, for those that persist or enlarge despite conservative measures, and for all male patients regardless of lesion size [2,10-11]. Liver transplant is reserved as a last resort for patients with tumors that occupy most of the liver, confirmed malignancy, or progression despite prior surgical resection [4].

In this case, following the initial diagnostic laparoscopy, further surgical intervention was not indicated, given the lesion’s size and the patient’s hemodynamic stability. Embolization was also deferred due to the potential risk of additional complications. The patient was advised to discontinue OCP use and to follow up with interval imaging. Unlike most reported cases, this patient’s lesion did not regress despite conservative management. If subsequent imaging shows no change in size, surgical intervention may be considered. The diameter of HAs tends to decrease significantly following menopause due to reduced estrogen stimulation. This decline in estrogen typically reduces the risk of rupture, making this patient's complication particularly unusual [6]. This case emphasizes the importance of individualized management by highlighting that serious complications of HA, particularly hemorrhage, can occur even in patients who do not meet typical risk criteria.

## Conclusions

This case highlights a potentially fatal presentation of a rare condition in a postmenopausal female, rupture of HA. It demonstrates that this complication can occur despite the expected regression of HA after menopause. Clinicians should maintain a high index of suspicion for HA rupture in older females, especially when imaging findings align with potential risk factors.
